# Differences in Leukocyte Telomere Length between Coronary Heart Disease and Normal Population: A Multipopulation Meta-Analysis

**DOI:** 10.1155/2019/5046867

**Published:** 2019-05-06

**Authors:** Xiaofeng Xu, Haochang Hu, Ying Lin, Fangzhong Huang, Huihui Ji, Yin Li, Shaoyi Lin, Xiaomin Chen, Shiwei Duan

**Affiliations:** ^1^Medical Genetics Center, School of Medicine, Ningbo University, Ningbo 315000, Zhejiang, China; ^2^Key Laboratory of Ningbo First Hospital and Cardiovascular Center of Ningbo First Hospital, Ningbo University, Ningbo 315000, Zhejiang, China

## Abstract

Coronary heart disease (CHD) is one of the most common causes of death in the world. Numerous studies have shown that as the degree of atherosclerotic disease increases, leukocyte telomere length gradually decreases. Short telomeres increase the risk of all-cause death and cardiovascular death. However, the reported results are not consistent, since the experimental design method, the measurement method, and the disease outcome are different. Therefore, we searched five major literature databases (Pubmed, Web of science, Embase, CNKI, and Wangfang) and finally included 18 eligible articles (including 5,150 patients with CHD and 9341 controls). We found that telomere length in patients with CHD was significantly shorter than that in controls, and the telomere length was inversely correlated with the severity of CHD. Subgroup analysis showed that telomere shortening was the most significant in Asian patients with CHD, in CHD patients with an average age <65 years, and in men with CHD. The mechanism of shortening the telomere length leading to the occurrence and development of CHD is worthy of further study.

## 1. Introduction

Coronary heart disease (CHD) is also known as coronary artery disease (CAD) and ischemic heart disease (IHD). CHD is the most common cause of human deaths worldwide [1]. The pathogenesis of CHD is complex, and hypertension, hyperlipidemia, smoking, obesity, and family history of CHD are risk factors for CHD [2]. Early detection of risk factors associated with CHD not only helps to reduce cardiovascular mortality, but also reduces the social medical burden.

Telomeres are TTAGGG nucleoprotein complexes repeats at the ends of eukaryotic chromosomes and are protective caps for chromosomes that support genomic integrity and stability. Telomere gradually shrinks with each cell division and is one of the hallmarks of aging [3]. Telomere length has an impact on various diseases, including cardiovascular diseases and cancer [4]. The extent of telomere loss is related to age and adverse lifestyle factors such as stress, smoking, and obesity [5], which prompts the potential correlation between CHD and telomere length. Lots of studies have shown that individuals with varying degrees of atherosclerosis have significantly different telomere lengths in their leukocyte [6, 7]. At the same time, telomere length is closely related to the risk of all-cause death and cardiovascular death in patients with CHD [8].

**Figure 1:**
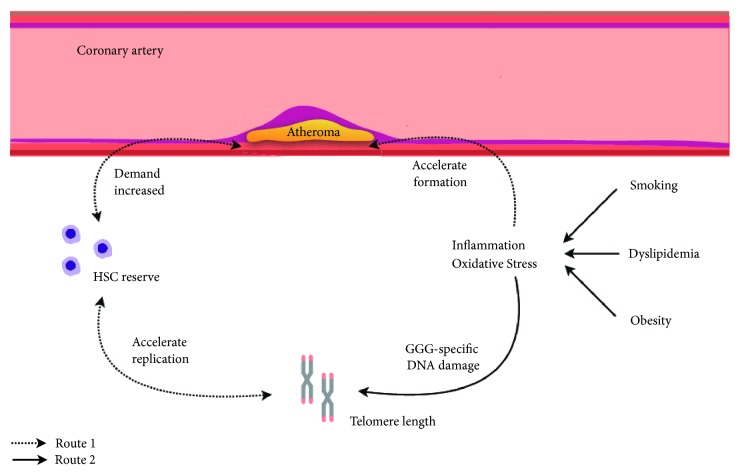
Overall model linking the telomere length with inflammation and oxidative stress. Route 1: in CHD patients, inflammation and oxidative stress accelerate the formation of atheroma. In order to repair the endothelial injury caused by atheroma, hematopoietic stem cells (HSC) will accelerate the replication in order to maintain its own reserve, accompanied by the shortening of telomeres in the cells. At the same time, telomere shortening will feed back to HSC, resulting in a decrease in replication rate and a decrease in their reserve. Atheroma becomes difficult to repair and more unstable. Route 2: oxidative stress and inflammation can use oxygen free radical (such as O^2−^) to increase the rate of telomere shortening by directly acting on the GGG-specific sequence in the telomere.

There is a commonly accepted opinion that CHD and its related risk factors can cause changes in telomere length (see Figure 1). The main conclusions are as follows: (1) For CHD patients, inflammatory reaction and oxidative stress may occur in the lesion site such as coronary atherosclerotic plaque. In order to cope with the above situation, the proliferation of hematopoietic stem cells (HSC) will speed up and this leads to faster shortening of telomeres [9, 10]. (2) Risk factors of CHD (such as smoking and obesity) will directly reduce the telomere length through increased systemic inflammatory and oxidative stress. The fact that low level of plasma trans fatty acids concentrations or high level of serum lipophilic antioxidants can delay telomere shortening also proves that inflammation and oxidative stress play an important role in regulating telomere length [11, 12].

Although many studies have attempted to clarify the relationship between telomere and cardiovascular disease, the results of these studies were inconsistent due to the differences in the experimental design methods, assays, and disease outcomes [13–15]. In the present study, we focused on the telomere studies with CHD as the only disease outcome. Our meta-analysis aimed to establish the relationship between telomere length and CHD and to provide a reference for the development of prevention and treatment strategies for CHD.

## 2. Materials and Methods

### 2.1. Literature Search Strategy

This meta-analysis was performed according to the MOOSE guidelines, and we aimed to establish the link between LTL and CHD. We searched the literatures in Pubmed, Web of science, Embase, CNKI, and Wangfang databases, and the deadline was February 12, 2019. Search terms included “telomere” (or “telomeres” or “telomeric” or “T/S ratio” or “T/C ratio”) and “cardiovascular diseases” (or “cardiovascular disease” or “vascular diseases” or “vascular disease” or “ischemic heart disease” or “myocardial ischaemia” or “myocardial ischemia” or “acute coronary syndrome” or “coronary disease” or “coronary heart disease” or “coronary artery disease” or “coronary occlusion” or “coronary stenosis” or “coronary artery stenosis” or “coronary thrombosis” or “myocardial infarction” or “heart attack”) in the title or abstract.

### 2.2. Selection Criteria

After initial screening, two authors (XU and HU) reviewed the full text and selected eligible researches based on established inclusion criteria. The inclusion criteria of eligible studies were as follows: (1) the study should use the definition and diagnosis of CHD in the 2014 Epidemiological studies of CHD [16, 17]; (2) the study subjects should include the CHD cases and the normal controls; (3) the study should include the mean and standard deviation of telomere length in both the CHD cases and the controls. In addition, we excluded the study containing incomplete experimental data.

### 2.3. Date Extraction

Authors (Lin and Huang) extracted data from each study included the following item: first author, reference, year of publication, country of study, population source, study design (prospective studies or retrospective study), PMID, participants' characteristics (n, mean age, proportion of male participants, race), case definition, assay method (qPCR, southern blotting of FISH) and TL unit, relative risk, and mean and standard deviation of TL in CHD or control group.

### 2.4. Quality Assessment

The methodological evaluation of the included literatures was based on the Newcastle-Ottawa Scale (NOS) assessment method. The highest score is 9 points [18]. Mainly based on the following three aspects, it ensured the minimum risk of bias: (1) study population selection (4 points); (2) intergroup comparability (2 points); (3) the exposures and outcomes for case-control and cohort studies (3 points). Two authors (XU and HU) assessed the NOS included in the study.

### 2.5. Statistical Analysis

All analyses were performed using the Stata (version 15.0) and the Review Manager (version 5.3) programs. We assessed the heterogeneity between studies using the Cochran Q test and I^2^ statistic. If I^2^ < 50%, the meta-analysis study could be considered homogeneous and use a fixed effect model; if I^2^ > 50%, a random effect model was used for meta-analysis. Subgroup analysis was performed based on possible heterogeneity factors, and sensitivity analysis was used to analyze the stability of the test results. Statistical tests were bilateral and P < 0.05 was considered statistically significant.

### 2.6. Publication Bias

Published bias assessments were demonstrated by funnel plots and Egger linear regression plots to assess the source of heterogeneity.

### 2.7. Subgroup Analysis

To address heterogeneity among study populations, subgroup analyses were performed as follows: Asia, Europe, or North America; prospective study vs. retrospective study; population-based study vs. other study; study quality < 7 vs. study quality ≥ 7; mean age < 65 vs. mean age ≥ 65; qPCR vs. other assay methods; the proportion of males <50% vs. the proportion of males ≥ 50%; case definition is fatal, nonfatal, or mixed.

## 3. Results

**Figure 2:**
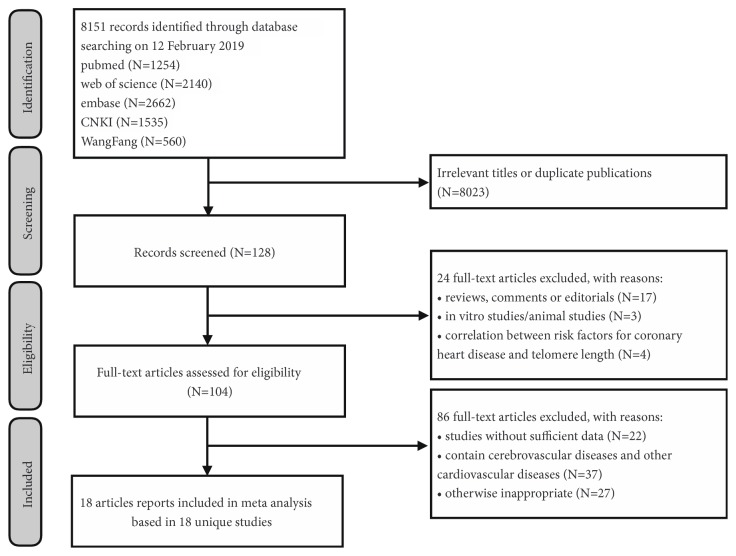
Flow diagram of eligible studies selection in present meta-analysis.

In the current study, a total of 5 major literature databases were searched, and a total of 8151 related articles were retrieved (see Figure 2). By reading the relevant headlines and abstracts, we excluded the publications with unrelated subjects and duplicated data. We further excluded reviews, editorials, animal experiments, and literatures on the risk factors of CHD with telomere length. We finally identified 18 eligible articles into our meta-analysis (see Table 1).

**Figure 3:**
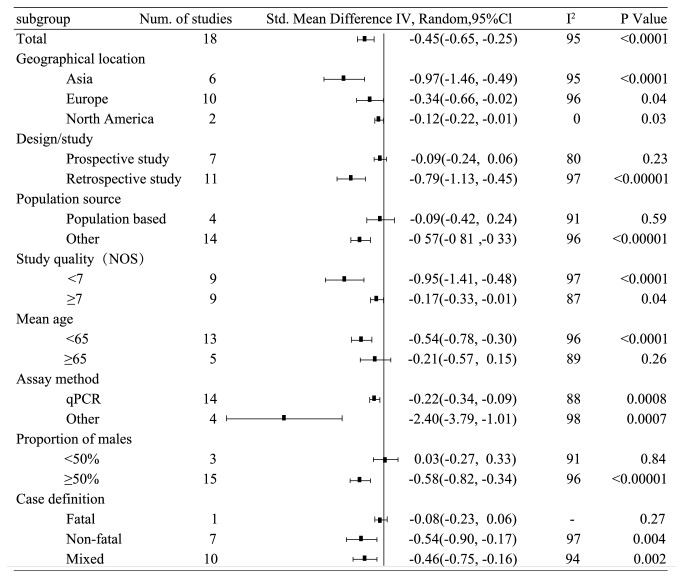
Standard mean difference of telomere length between CHD and control group by recorded study level characteristics.

Overall, the meta-analysis involved 14491 individuals, including 5150 CHD cases and 9341 controls. The average age of all participants was 62 years and the male ratio was 58.5%. In terms of regional distribution, there were 6 studies in Asia (5231 participants), 10 studies in Europe (5676 participants), and 2 studies in North America (3584 participants). And there were 7 prospective studies and 11 retrospective studies. There were 4 population-based studies and 14 non-population-based studies. There were 13 studies with an average age of < 65 and 5 studies with an average age of ≥ 65. Fourteen studies used qPCR to measure telomere length, and four studies used Southern blot or flow-FISH to measure telomere length. At the same time, we performed a quality check on all included studies using the Newcastle-Ottawa Scale score, and we found there were 9 studies with a score of ≥ 7 and 9 studies with a score of < 7 (see Figure 3).

### 3.1. Correlation between Telomere Length and CHD

**Figure 4:**
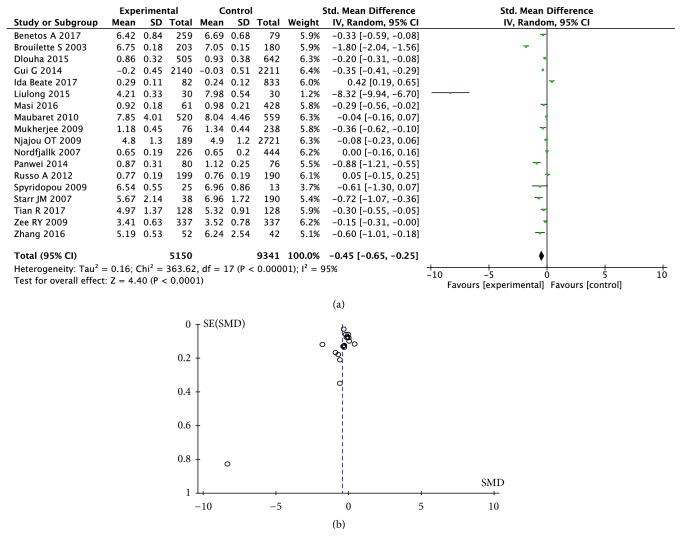
Forest plot (a) and funnel plot (b) of telomere length in CHD. SD: standard difference; SMD: standard mean difference.

In the meta-analysis of all the 18 studies, the LTL in CHD patients was significantly shorter than that in the controls (standard mean difference (SMD) = -0.45; 95% CI (-0.65, -0.25), P < 0.0001; see Figure 4.) As shown in the Figure 3, among the studies in Asia, Europe, and North America, the association of telomere length with the risk of CHD was most significant in Asia (SMD = -0.97; 95% CI (-1.46, -0.49), P < 0.0001; see Supplemental Figure 1). In a retrospective study, telomere length was also significantly shorter in CHD patients than in the controls (SMD = -0.79; 95% CI (-1.13, -0.45), P < 0.0001; see Supplemental Figure 2). In a prospective study, there was no significant difference of telomere length between the CHD patients and the controls (SMD = -0.09 95% CI (-0.24, 0.06), P = 0.23; see Supplemental Figure 2). Fourteen non-population-based (hospital-derived) studies showed that telomere length was significantly shorter in CHD patients than in the controls (SMD = -0.57; 95% CI (-0.81, -0.33), P < 0.00001; see Supplemental Figure 3), but there was no significant association of shortened telomere length with CHD in 4 population-based studies (SMD = -0.09; 95% CI (-0.42, -0.24), P = 0.59; see Supplemental Figure 3). According to the study quality, we found the significant difference between telomere length of CHD patients in studies with high quality scores (NOS ≥ 7) (SMD = -0.17, 95% CI (-0.33, -0.01), P = 0.04; see Supplemental Figure 4) and those in studies with low quality scores (NOS < 7) (SMD = -0.95, 95% CI (-1.41, -0.48), P < 0.0001; see Supplemental Figure 4). In the study with a mean age of participants ≥ 65 years, there was no significant difference in telomere length between CHD cases and controls (SMD = -0.21 95% CI (-0.57, 0.51), P = 0.26; see Supplemental Figure 5). In the study of participants with an average age < 65 years, the telomere length of CHD patients was significantly shorter than that of the controls (SMD = -0.54; 95% CI (-0.78, -0.30), P < 0.0001; see Supplemental Figure 5). We also found significant differences of telomere length between CHD patients and the controls in the studies using both qPCR method and other methods (qPCR: SMD = -0.22, 95% CI (-0.34, -0.09), P = 0.0008 and other methods: SMD = -2.40, 95% CI (-3.79, -1.01), P = 0.0007; see Supplemental Figure 6). In the studies with ≥50% male participants, patients with CHD had shorter telomere length (SMD = -0.58; 95% CI (-0.82, -0.34), P < 0.00001; see Supplemental Figure 7). In the studies with a male participant ratio < 50%, there was no significant difference in telomere length between the CHD cases and the controls (SMD = 0.03, 95% CI (-0.27, 0.33), P = 0.84; see Supplemental Figure 7). When the outcome of the experimental group was death in CHD patients, there was no significant difference in telomere length between CHD cases and controls (SMD = -0.08, 95% CI (-0.27, 0.33), P = 0.27; see Supplemental Figure 8). When the outcome of the experimental group was mixed or nonfatal, the telomere length in CHD patients was significantly shorter than the control group (mixed: SMD = -0.46, 95% CI (-0.75, -0.16), P = 0.002 and nonfatal SMD = -0.54, 95% CI (-0.90, -0.17), P = 0.004; see Supplemental Figure 8).

### 3.2. Heterogeneity Analysis

There was significant heterogeneity among the studies in the meta-analysis (P < 0.00001, I^2^ = 95%). After subgroup analysis by human race (see Supplemental Figure 1), we found no significant heterogeneity in the North American studies (I^2^ = 0%), but heterogeneity was present in both Asian studies (I^2^ = 95%) and in European studies (I^2^ = 96%). We also performed subgroup meta-analyses by various parameters, including design methods, population, article quality score, age, experimental methods, gender ratio, and outcome of the experimental group (see Supplemental Figures 2-8). Significant heterogeneity was present in all the subgroup meta-analyses (I^2^ > 50%). Metaregression analysis showed that all the above factors did not decrease the heterogeneity (p > 0.05). Our findings suggested that the heterogeneity of the current meta-analysis might be only related to human race.

### 3.3. Sensitivity Analysis

Sensitivity analysis showed that the results of the studies of Broililette S [19], Ida Beate [14], and Liu Long [20] had the greatest influence on the difference of telomere length between the CHD cases and the controls. Moreover, a second heterogeneity test had little effect on the results, suggesting that our meta-analysis had certain stability, and it was not necessary to eliminate the studies with large heterogeneity.

### 3.4. Publication Bias

**Figure 5:**
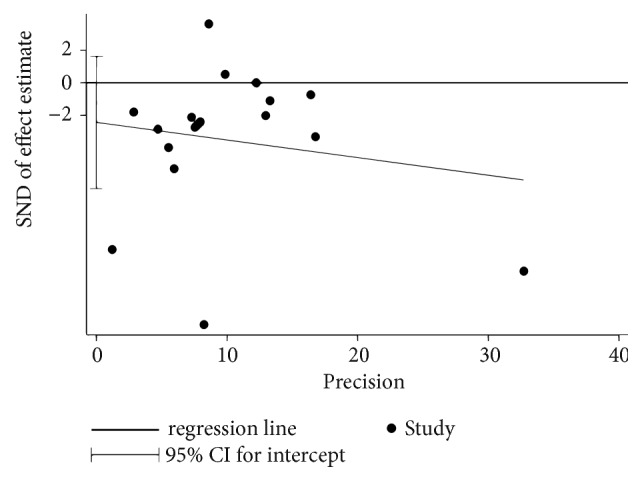
Egger tests for the assessment of publication bias of telomere length in CHD. SND: Standard.

The funnel plot showed that the 18 studies were concentrated and there was no obvious asymmetric distribution (see Figure 4(b)). The Egger regression analysis showed there was no publication bias (t = 1.27, P = 0.224; see Figure 5).

## 4. Discussion

There are related studies showing that LTL is significantly associated with the development of CHD [21–23]. Telomere length predicts mortality in patients with stable CHD [24] and is also associated with coronary atherosclerosis [25]. In addition, longer telomeres were associated with lower prevalence of subclinical atherosclerosis and peripheral arterial disease [15]. Through systematic analysis of 18 articles in the current study, the telomere length of patients with CHD was significantly shorter than that of controls, and the telomere length was inversely correlated with the severity of CHD. Subgroup analyses showed that telomere shortening was the most significant in Asian patients with CHD, in the patients with CHD with an average age < 65 years, and in the male patients with CHD.

Previous studies showed there were cases of shortening of telomere length in patients with CHD in both Caucasians [26, 27] and Asians [28, 29]. A study examined the LTL by 1525 postmenopausal women and indicated that LTL was closely related to human race. Specifically, African American telomere length was longer than whites [30]. In this study, we found that in Asians, telomere shortening was evident in patients with CHD. This indicates that there is a difference in the changes in telomere length between patients with CHD among different races.

Previous studies have shown that the prevalence and age of onset of CHD were different between men and women, while the age of onset of CHD was usually 10 years later than men [31]. Our meta-analysis found that in the studies of male ratio less than 50%, the difference in LTL between CHD patients and normal control group was not significant, but the LTL difference was significant in the studies of male ratio ≥50%. In a study investigating the relationship between LTL and adult American heart risk, the mean serum high-density lipoprotein cholesterol, average fat mass, fat-free, hemoglobin A 1c (HbA 1c), and C-reactive protein were significantly reduced as telomere length increases in men. However, there was only a decrease in HbA1c as telomere length increases in women [32]. Therefore, gender may also be one of the factors related to telomere length affecting CHD, but further research is needed to confirm this conclusion.

In studies with an average age of ≥ 65 years and death in patients with CHD, the difference in LTL between CHD cases and controls was not significant. A recent study suggested that longer LTL increased the proportion of myocardial infarction in both males and females when the baseline age was ≥ 65 [14]. This may be related to the different roles of telomere shortening in various stages of atherosclerosis. It has been reported that telomere shortening was involved in advanced vascular disease and acute vascular syndrome [33]. Therefore, the relationship between telomere length and CHD needs to be further explored in people with age baseline ≥ 65 and fatal CHD. In addition, our subgroup analysis showed that telomere shortening was the most significant in the patients with CHD with an average age < 65 years. However, more experiments need to be performed to confirm our findings.

We also found an inverse correlation between the severity of CHD and LTL. Related studies have reported that the more the atherosclerotic plaques, the shorter the telomere length [6]. We found a significant difference in LTL between CHD patients and controls in 14 nonpopulation (hospital-derived) studies, whereas the difference in LTL between CHD patients and controls in the two population-based studies was not significant. This may be because hospital-derived patients with CHD have different severity of disease among the CHD patients.

There are several strengths of the current study. This article contains 18 studies from January 2003 to February 2019, and our meta-analysis compared the results of studies in Asia, Europe, and North America. Excluding the effects of heart failure, stroke, and other cardiovascular diseases, the relationship between CHD and telomere length was precisely defined. All the included studies were normal-controlled trials with acceptable methodological quality and the least probable chance of bias. However, there are several potential limitations in this study. Firstly, since the enrolled studies were mainly observational studies, the relationship between telomere length and CHD may be affected by residual confounding or reverse causality. Therefore, the causal link between telomere length and CHD could not be inferred. CHD is a multifactorial disease that involves several complex genetic and environmental factors. We cannot completely eliminate the potential impact of environmental factors on the results. Therefore, the interaction of genetic and environmental factors in the development of CHD needs to be confirmed in further studies of larger and diverse samples. Secondly, the heterogeneity is still large in the subgroup meta-analyses. Although we speculate that it comes from human race, we still could not confirm the source of related heterogeneity. Thirdly, the degree of inverse correlation between CHD and telomere length was different among various human races. More ethnic groups should be included in the study in order to draw scientific conclusions.

In summary, the shortening of telomere length was significantly associated with the risk of CHD. The telomere length in nonfatal male CHD cases younger than 65 years was significantly shorter than in controls. At the same time, we also found that the more severe the CHD, the more severe the telomere wear. However, telomere length is related to age and ethnicity, and each person has significant individual differences at birth. Therefore, we need more prospective study to observe changes about telomere length after CHD is diagnosed. And more cross-ethnic multicenter research is needed. In the future, the mechanism of shortening telomere length leading to the occurrence and development of CHD is worthy of further study.

## Figures and Tables

**Table 1:** Selected characteristics of 18 studies in review of leucocyte telomere length and CHD.

P/R studies	Major investigators	NOS	Mean TL in CHD(SD)	CHD cases	Mean TL in Control (SD)	Control case	Location	Year	Ethnicity	Population source	Mean age (SD)	Men (%)	Total cases	Assay method	CHD definition	TL units
Prospective studies	Zee RY	8	3.41(0.63)	337	3.52(0.78)	337	US	2009	white American	Occupational list	60(9)	100	674	qPCR	mixed	loge T/S

Prospective studies	Ida Beate	8	0.29(0.11)	82	0.24(0.12)	833	Norway	2017	Norwegian	Population based cohort	71(5)	48	915	qPCR	mixed	T/S

Prospective studies	Nordfjallk	7	0.65(0.19)	226	0.65(0.20)	444	Sweden	2007	Swede	Population based cohort	61(5)	69	670	qPCR	mixed	T/S

Prospective studies	Njajou OT	7	4.80(1.30)	189	4.90(1.20)	2721	US	2009	African American and white American	Insurance register and area code residence	74(3)	49	2910	qPCR	fatal	kb

Retrospective studies	Cui G	7	-0.20(0.45)	2140	-0.03(0.51)	2211	China	2014	Chinese	Hospital patients/community sample	59(9)	54	4351	qPCR	non-fatal	loge T/S

Retrospective studies	Zhang	7	5.19(0.53)	52	6.24(2.54)	42	china	2016	Chinese	Hospital patients/volunteers	49(9)	60	94	qPCR	mixed	kb

Prospective studies	Masi	7	0.92(0.18)	61	0.98(0.21)	428	UK	2016	British	Hospital patients/volunteers	67(27,91)	62	489	qPCR	mixed	T/S

Prospective studies	Benetos A	7	6.42(0.84)	259	6.69(0.68)	79	France	2017	French, Athenian	Hospital patients/volunteers	63(14)	68	338	Southern blotting	mixed	kb

Retrospective studies	Tian R	7	4.97(1.37)	128	5.32(0.91)	128	China	2017	Chinese	Hospital patients/volunteers	49(7)	74	256	qPCR	mixed	kb

Retrospective studies	Maubaret	6	7.85(4.01)	520	8.04(4.46)	559	Multiple	2010	European	Hospital patients/community sample	52(6)	100	1079	qPCR	non-fatal	loge T/S

Retrospective studies	Russo A	6	0.77(0.19)	199	0.76(0.19)	190	Italy	2012	Italian	Hospital patients/unspecified	40(5)	90	389	qPCR	non-fatal	T/S

Retrospective studies	Brouilette S	5	6.75(0.18)	203	7.05(0.15)	180	UK	2003	British	Hospital register/GP register	47(6)	86	383	Southern blotting	non-fatal	kb

Retrospective studies	Starr JM	5	5.67(2.14)	38	6.96(1.72)	190	UK	2007	British	Population based cohort	79(0)	82	228	qPCR	non-fatal	kb

Retrospective studies	Mukherjee	5	1.18(0.45)	76	1.34(0.44)	238	India	2009	Indian	Hospital patients/community sample	57(11)	78	314	qPCR	non-fatal	T/S

Retrospective studies	panweiwei	5	0.87(0.31)	80	1.12(0.25)	76	china	2014	Chinese	Hospital patients	58(4)	53	156	qPCR	mixed	T/S

Retrospective studies	Liulong	4	4.21(0.33)	30	7.98(0.54)	30	China	2015	Chinese	Hospital patients/volunteers	47(6)	67	60	Southern blotting	mixed	kb

Prospective studies	Dlouha	4	0.86(0.32)	505	0.93(0.38)	642	Czech	2015	Czech	Insurance register/Hospital patients	60(7)	0	1147	qPCR	mixed	T/S

Retrospective studies	Spyridopoulos I	3	6.54(0.55)	25	6.96(0.86)	13	Germany	2009	German	Hospital patients/volunteers	65(2)	100	38	FISH	non-fatal	kb

CHD: coronary heart disease; FISH: flow cytometry-fluorescent in situ hybridization; NOS: Newcastle-Ottawa scale; qPCR: quantitative polymerase chain reaction.

## Supplementary Materials

Supplementary Materials In Supplemental Figures, we roundly evaluated the association of telomere length shorten with CHD in different subgroup. All the figures showed forest plots of telomere length in CHD grouped by race (Supplemental Figure 1), the prospective study or retrospective study (Supplemental Figure 2), the population source (Supplemental Figure 3), the study quality (Supplemental Figure 4), mean age of patients (Supplemental Figure 5), the assay method (Supplemental Figure 6), the proportion of male participants (Supplemental Figure 7), and the case definition (Supplemental Figure 8), respectively. And I have added more detailed descriptions of each supplementary material in the e-mail attachment. The word named “The Search Strategy” describes in detail the retrieval conditions of each database.
