# Supplementary material for: Differences in Leukocyte Telomere Length between Coronary Heart Disease and Normal Population: A Multipopulation Meta-Analysis
Source: Biomed Res Int. 2019 May 6;2019:5046867. doi: 10.1155/2019/5046867 (PMC6526555; doi:10.1155/2019/5046867)
Supplement: Supplementary Materials — In Supplemental Figures, we roundly evaluated the association of telomere length shorten with CHD in different subgroup. All the figures showed forest plots of telomere length in CHD grouped by race (Supplemental Figure 1), the prospective study or retrospective study (Supplemental Figure 2), the population source (Supplemental Figure 3), the study quality (Supplemental Figure 4), mean age of patients (Supplemental Figure 5), the assay method (Supplemental Figure 6), the proportion of male participants (Supplemental Figure 7), and the case definition (Supplemental Figure 8), respectively. And I have added more detailed descriptions of each supplementary material in the e-mail attachment. The word named “The Search Strategy” describes in detail the retrieval conditions of each database. [file 5046867.f1.zip › The Search Strategy (1).docx]

**Differences in leukocyte telomere length between coronary heart disease and normal population: a multi-population meta-analysis**

Xiaofeng Xu1, Haochang Hu1, Ying Lin1, Fangzhong Huang1, Huihui Ji1, Yin Li1, Shaoyi Lin2, Xiaomin Chen2*, Shiwei Duan1*

1. Medical Genetics Center, School of Medicine, Ningbo University, Ningbo, Zhejiang, China 315000

2. Key Laboratory of Ningbo First Hospital and Cardiovascular Center of Ningbo First Hospital, Ningbo University, Ningbo, Zhejiang China 315010

*: Correspondence should be addressed to Dr. Shiwei Duan (duanshiwei@nbu.edu.cn) and Dr. Xiaomin Chen (chxmin@hotmail.com).

The Search Strategy

**PubMed, search through 12 February 2019 (total 1254)**

("Telomere"[Mesh] OR "Telomere" OR "Telomeres" OR "telomeric" OR "T/S ratio" OR "T/C ratio") AND ("Cardiovascular Diseases"[Mesh] OR "Cardiovascular Diseases" OR "Cardiovascular Disease" OR "Vascular Diseases" OR "Vascular Disease" OR "Ischemic Heart Disease" OR "Myocardial ischaemia" OR "Myocardial ischemia" OR "Acute coronary syndrome" OR "Coronary disease" OR "Coronary heart disease" OR "Coronary artery disease" OR "Coronary occlusion" OR "Coronary stenosis" OR "Coronary artery stenosis" OR "Coronary thrombosis" OR "Myocardial infarction" OR "Heart attack")

**Web of Science, search through 12 February 2019 (total 2140)**

TS=("Telomere" OR "Telomeres" OR "telomeric" OR "T/S ratio" OR "T/C ratio") AND TS=("Cardiovascular Diseases" OR "Cardiovascular Disease" OR "Vascular Diseases" OR "Vascular Disease" OR "Ischemic Heart Disease" OR "Myocardial ischaemia" OR "Myocardial ischemia" OR "Acute coronary syndrome" OR "Coronary disease" OR "Coronary heart disease" OR "Coronary artery disease" OR "Coronary occlusion" OR "Coronary stenosis" OR "Coronary artery stenosis" OR "Coronary thrombosis" OR "Myocardial infarction" OR "Heart attack")

**EMBASE, search through 12 February 2019 (total 2662)**

("Telomere" OR "Telomeres" OR "telomeric" OR "T/S ratio" OR "T/C ratio") AND ("Cardiovascular Diseases" OR "Cardiovascular Disease" OR "Vascular Diseases" OR "Vascular Disease" OR "Ischemic Heart Disease" OR "Myocardial ischaemia" OR "Myocardial ischemia" OR "Acute coronary syndrome" OR "Coronary disease" OR "Coronary heart disease" OR "Coronary artery disease" OR "Coronary occlusion" OR "Coronary stenosis" OR "Coronary artery stenosis" OR "Coronary thrombosis" OR "Myocardial infarction" OR "Heart attack")

**CNKI, search through 12 February 2019 (total 1535)**

FT=("Telomere" OR "Telomeres" OR "telomeric" OR "T/S ratio" OR "T/C ratio") AND FT=（"Cardiovascular Diseases" OR "Cardiovascular Disease" OR "Vascular Diseases" OR "Vascular Disease" OR "Ischemic Heart Disease" OR "Myocardial ischaemia" OR "Myocardial ischemia" OR "Acute coronary syndrome" OR "Coronary disease" OR "Coronary heart disease" OR "Coronary artery disease" OR "Coronary occlusion" OR "Coronary stenosis" OR "Coronary artery stenosis" OR "Coronary thrombosis" OR "Myocardial infarction" OR "Heart attack"）

**WANGFANG, search through 12 February 2019 (total 560)**

Theme: ("Telomere" OR "Telomeres" OR "telomeric" OR "T/S ratio" OR "T/C ratio") AND Theme:（"Cardiovascular Diseases" OR "Cardiovascular Disease" OR "Vascular Diseases" OR "Vascular Disease" OR "Ischemic Heart Disease" OR "Myocardial ischaemia" OR "Myocardial ischemia" OR "Acute coronary syndrome" OR "Coronary disease" OR "Coronary heart disease" OR "Coronary artery disease" OR "Coronary occlusion" OR "Coronary stenosis" OR "Coronary artery stenosis" OR "Coronary thrombosis" OR "Myocardial infarction" OR "Heart attack"）
