# Supplementary material for: Differences in Leukocyte Telomere Length between Coronary Heart Disease and Normal Population: A Multipopulation Meta-Analysis
Source: Biomed Res Int. 2019 May 6;2019:5046867. doi: 10.1155/2019/5046867 (PMC6526555; doi:10.1155/2019/5046867)
Supplement: Supplementary Materials — In Supplemental Figures, we roundly evaluated the association of telomere length shorten with CHD in different subgroup. All the figures showed forest plots of telomere length in CHD grouped by race (Supplemental Figure 1), the prospective study or retrospective study (Supplemental Figure 2), the population source (Supplemental Figure 3), the study quality (Supplemental Figure 4), mean age of patients (Supplemental Figure 5), the assay method (Supplemental Figure 6), the proportion of male participants (Supplemental Figure 7), and the case definition (Supplemental Figure 8), respectively. And I have added more detailed descriptions of each supplementary material in the e-mail attachment. The word named “The Search Strategy” describes in detail the retrieval conditions of each database. [file 5046867.f1.zip › figures.doc]

**Differences in leukocyte telomere length between coronary heart disease and normal population: a multi-population meta-analysis**

**Supplemental Figure**

Xiaofeng Xu^1^, Haochang Hu^1^, Ying Lin^1^, Fangzhong Huang^1^, Huihui Ji^1^, Yin Li^1^, Shaoyi Lin^2^, Xiaomin Chen^2^*, Shiwei Duan^1^*

1. Medical Genetics Center, School of Medicine, Ningbo University, Ningbo, Zhejiang, China 315000
2. Key Laboratory of Ningbo First Hospital and Cardiovascular Center of Ningbo First Hospital, Ningbo University, Ningbo, Zhejiang China 315010

*: Correspondence should be addressed to Dr. Shiwei Duan (duanshiwei@nbu.edu.cn) and Dr. Xiaomin Chen (chxmin@hotmail.com).

**Supplemental Figure 1:** Forest plot of telomere length in CHD grouped by race.

In Asia studies, telomere length was shorter in CHD patients than in the controls (SMD = -0.97; 95% CI (-1.46, -0.49), P < 0.0001). In Europe studies, telomere length was shorter in CHD patients than in the controls (SMD = -0.34; 95% CI (-0.66, -0.02), P = 0.04). And in North America studies, telomere length was also shorter in CHD patients than in the controls (SMD = -0.12; 95% CI (-0.22, -0.01), P = 0.03).

**Supplemental Figure 2:** Forest plot of telomere length in CHD grouped by the prospective study or retrospective study.

In prospective studies, there was no significant difference of telomere length between the CHD patients and the controls (SMD = -0.09; 95% CI (-0.24, 0.06), P = 0.23). In retrospective studies, telomere length was significantly shorter in CHD patients than in the controls (SMD = -0.79; 95% CI (-1.13, -0.45), P < 0.0001).

**Supplemental Figure 3:** Forest plot of telomere length in CHD grouped by the population source.

In population-based studies, there was no significant difference of telomere length between the CHD patients and the controls (SMD = -0.09; 95% CI (-0.42, -0.24), P = 0.59). But in non-population-based (hospital-derived) studies, telomere length was significantly shorter in CHD patients than in the controls (SMD = -0.57; 95% CI (-0.81, -0.33), P < 0.00001).

**Supplemental Figure 4:** Forest plot of telomere length in CHD grouped by the study quality.

In low quality scores (NOS < 7) studies, telomere length was significantly shorter in CHD patients than in the controls (SMD = -0.95, 95% CI (-1.41, -0.48), P < 0.0001). And in high quality scores (NOS ≥ 7) studies, telomere length was also significantly shorter in CHD patients than in the controls (SMD = -0.17, 95% CI (-0.33, -0.01), P = 0.04).

**Supplemental Figure 5:** Forest plot of telomere length in CHD grouped by mean age of patients.

In the study of participants with an average age < 65 years, the telomere length of CHD patients was significantly shorter than that of the controls (SMD = -0.54; 95% CI (-0.78, -0.30), P < 0.0001). In the study with a mean age of participants ≥ 65 years, there was no significant difference in telomere length between CHD cases and controls (SMD = -0.21 95% CI (-0.57, 0.51), P = 0.26).

**Supplemental Figure 6:** Forest plot of telomere length in CHD grouped by the assay method.

In the studies using qPCR method, the telomere length of CHD patients was significantly shorter than that of the controls (SMD = -0.22, 95% CI (-0.34, -0.09), P = 0.0008). In the studies using other methods, the telomere length of CHD patients was also significantly shorter than that of the controls (SMD = -2.40, 95% CI (-3.79, -1.01), P = 0.0007)

**Supplemental Figure 7:** Forest plot of telomere length in CHD grouped by the proportion of male participants.

In the studies with a male participant ratio <50%, there was no significant difference in telomere length between the CHD cases and the controls (SMD = 0.03, 95% CI (-0.27, 0.33), P = 0.84). In the studies with ≥50% male participants, patients with CHD had shorter telomere length (SMD = -0.58; 95% CI (-0.82, -0.34), P < 0.00001).

**Supplemental Figure 8:** Forest plot of telomere length in CHD grouped by the case definition.

When the outcome of the experimental group was death in CHD patients, there was no significant difference in telomere length between CHD cases and controls (SMD = -0.08, 95% CI (-0.23, 0.06), P=0.27). When the outcome of the experimental group was mixed or non-fatal, the telomere length in CHD patients was significantly shorter than the control group (non-fatal: SMD=-0.54, 95% CI (-0.90, -0.17), P = 0.004 and mixed: SMD = -0.46, 95% CI (-0.75, -0.16), P = 0.002).
